# LncRNA MT1JP functions as a tumor suppressor by interacting with TIAR to modulate the p53 pathway

**DOI:** 10.18632/oncotarget.7487

**Published:** 2016-02-19

**Authors:** Lihui Liu, Haiyan Yue, Qinghua Liu, Jiao Yuan, Jing Li, Guifeng Wei, Xiaomin Chen, Youyong Lu, Mingzhou Guo, Jianjun Luo, Runsheng Chen

**Affiliations:** ^1^ Key Laboratory of RNA Biology, Institute of Biophysics, Chinese Academy of Sciences, Beijing 100101, China; ^2^ Beijing Key Laboratory of Noncoding RNA, Institute of Biophysics, Chinese Academy of Sciences, Beijing 100101, China; ^3^ Graduate School of Chinese Academy of Sciences, Beijing 100049, China; ^4^ Institute of Basic Medical Sciences, Chinese Academy of Medical Sciences, Peking Union Medical College Hospital, Center of Excellence in Tissue Engineering, School of Basic Medicine, Peking Union Medical College, Beijing 100005, China; ^5^ Laboratory of Molecular Oncology, Key Laboratory of Carcinogenesis and Translational Research (Ministry of Education), Peking University Cancer Hospital and Institute, Beijing 100142, China; ^6^ Department of Gastroenterology and Hepatology, Chinese PLA General Hospital, Beijing 100853, China; ^7^ Research Network of Computational Biology, RNCB, Beijing 100101, China

**Keywords:** long noncoding RNAs, MT1JP, TIAR, p53, tumor suppressor

## Abstract

Accumulating evidence suggests that long noncoding RNAs (lncRNAs) play important roles in transcriptional regulation, whereas the extent to which the lncRNAs also function at the posttranscriptional level is less known. In the present study, we report a lncRNA named *MT1JP* which acts as a tumor suppressor through a posttranscriptional mechanism. We found that *MT1JP* is differentially expressed in tumor tissues by analyzing data from a customized microarray applied to 76 pairs of matched normal and cancer tissue samples. By associating with the RNA-binding protein TIAR, *MT1JP* enhanced the translation of the master transcription factor p53, thereby regulating a series of pathways involving p53, such as the cell cycle, apoptosis and proliferation. When *MT1JP* was down-regulated, the protein level of p53 declined, which in turn accelerated cell deterioration and tumor formation. Moreover, differential expression of *MT1JP* in cancerous and normal tissues suggests that it may be a promising prognostic marker and a therapeutic target. Taken together, we identified *MT1JP* as a critical factor in restraining cell transformation by modulating p53 translation through interactions with TIAR, and this finding is likely to shed new light on future investigations about posttranscriptional or translational effects of lncRNAs during cell transformation.

## INTRODUCTION

Long noncoding RNAs (lncRNAs) are defined as a large class of transcripts that are longer than 200 nt but lack protein-coding ability [1]. According to their genomic locations, lncRNAs can be categorized as follows: large intergenic noncoding RNAs (lincRNAs), intronic lncRNAs, antisense transcripts, bidirectional lncRNAs and pseudogenes [2]. LncRNAs have been shown to be involved in diverse cellular processes including the cell cycle and apoptosis [3–6], development and differentiation [7–9], X chromosome inactivation and gene imprinting [10–12]. Dysregulation of lncRNAs have been implicated in a number of diseases, including neurodegenerative diseases, cardiovascular diseases and cancer [7, 13–15]. Cancer is a group of complex diseases involving abnormal cell growth and tumorigenesis, and increasing evidence indicates that lncRNAs play key roles in cancer cells. However, lncRNA expression is often tissue-specific, and few lncRNAs with a consistent expression pattern in different types of cancer have been reported. One exception is the lncRNA *MALAT1*, whose expression is deregulated in various cancers. *MALAT1* was first shown to be over-expressed in hepatoblastomas compared to hepatocellular carcinomas [16], and later investigations have indicated that *MALAT1* is also upregulated in colon, breast, prostate and non-small cell lung cancer (NSCLC) [17–20].

The majority of lncRNAs regulate cellular processes at the epigenetic or transcriptional levels [21–24]. LncRNAs modulate DNA by affecting chromatin state as a tether, scaffold or in other roles while regulating gene transcription through monitoring the activity of specific transcription factors and polymerases. The most celebrated one is lincRNA *HOTAIR* which not only represses the *HOXD* locus gene transcription *in trans* but also regulates chromatin states and epigenetic inheritance by acting as a modular scaffold [21, 25]. However, little is known of the detailed mechanism by which lncRNAs exert their effects at the post-transcriptional level during tumorigenesis. *LincRNA-p21* exerts its repression of mRNA translation by interacting with HuR [26], but in general, there are few examples of how lncRNAs influence post-transcriptional processes such as alternative splicing and translation.

Here, we investigated *MT1JP*, a lncRNA that appears to play a key regulatory role upstream of p53. By examining its expression by microarray analysis, we discovered that *MT1JP* has a lower expression level in four types of cancerous tissues than in the corresponding normal tissues of liver, lung, colon or gaster. We further demonstrated that *MT1JP* affects the translational activity of the tumor suppressor p53 through interaction with TIAR, a RNA-binding protein which has not previously been reported to associate with lncRNAs. By acting as a p53 regulator, *MT1JP* modulates a series of cancer hallmarks in which p53 participates, ranging from cell cycle, apoptosis, proliferation to migration and invasion. Together, our results suggest that *MT1JP* is a novel tumor suppressor, and broaden our knowledge of the development of human tumors. More generally, its consistent expression pattern in numerous tumor specimens indicates that *MT1JP* may be a significant biomarker of diagnosis with a potential in cancer therapy.

## RESULTS

### lncRNA *MT1JP* has lower expression level in cancer than in normal tissue

**Figure 1:**
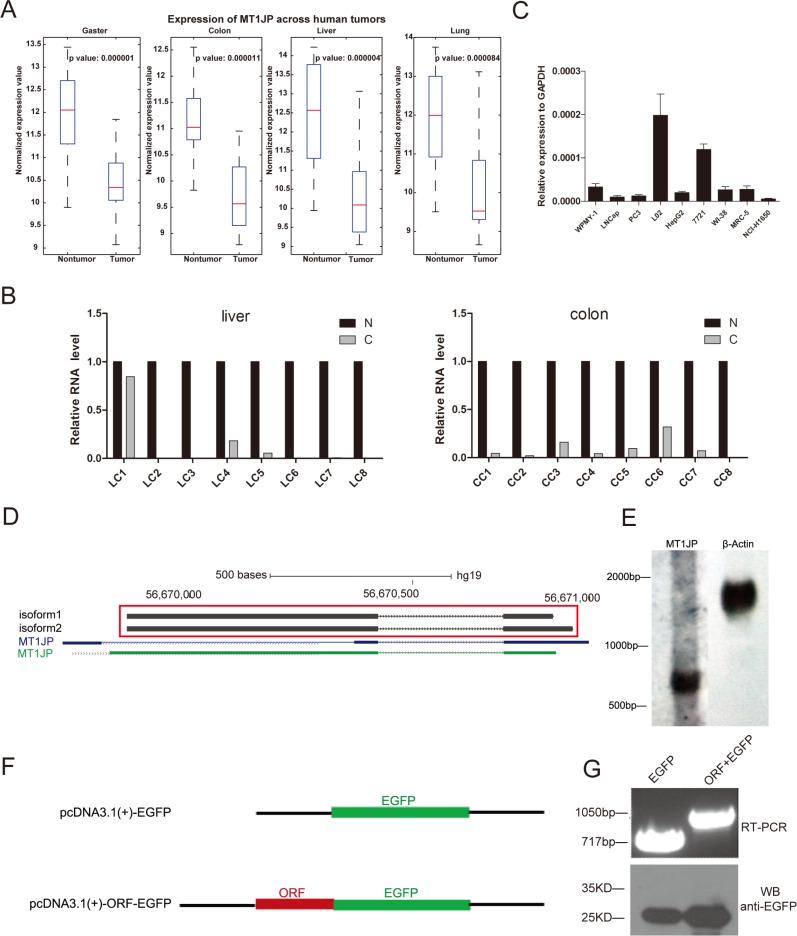
Identification of *MT1JP* as a tumor-associated lncRNA (**A**) Box plots showing *MT1JP* expression levels in tumor and paired adjacent nontumorous tissues from gaster (*n* = 20 × 2), colon (*n* = 20 × 2), liver (*n* = 16 × 2) and lung (*n* = 20 × 2). (**B**) Relative expression of *MT1JP* in additional 16 pairs of normal (N) and cancer (**C**) tissue samples from liver and colon were determined by qRT-PCR. (C) *MT1JP* expression in 9 human cell lines derived from 3 different tissues. (**D**) Two different isoforms of *MT1JP* were identified in the L02 cell line by 5′ and 3′ RACE. (**E**) Northern blot of RNA from the L02 cell line, indicating the *MT1JP* transcript size. *ACTB* is used as a control. (**F**) Left panel: Illustration of plasmids for coding potential verification. Right panel: Analysis of the transfected cells by RT-PCR (top) and Western blot (lower). The fusion transcript of *MT1JP* ORF and *EGFP* is detected, but the fusion protein is not.

To profile the transcriptomes of tumor tissues and investigate the roles of lncRNAs in tumorigenesis, we applied an mRNA + lncRNA custom-designed microarray probing more than 70,000 human transcripts to 76 pairs of cancerous and paracancerous tissue specimens from four types of cancer, including liver, lung, colon and gaster. Bioinformatics analysis showed that 157 lncRNAs were consistently differentially expressed between cancerous and adjacent benign tissue in all types of samples [27]. Among these, we observed one that was down-regulated to less than 50% in all tumor samples compared to the matched normal tissues (Figure 1A), and this transcript has previously been labelled as *MT1JP*. As an additional test, we determined the abundance of *MT1JP* in another 29 pairs of tissue samples from liver, colon, lung and gaster cancer by quantitative real time polymerase chain reaction (qRT-PCR). In line with the microarray analysis, we found that *MT1JP* had considerably lower expression in almost all tumor samples (Figure 1B and Supplementary Figure S1A). Moreover, the RNA-seq data from human body map showed that *MT1JP* was detected in kinds of tissues including liver, lung and others (Supplementary Figure S1B).

In order to select one or more cell lines for functional studies of this lncRNA, we examined the *MT1JP* expression in several cell lines derived from normal or cancer cells of three kinds of tissues (Figure 1C). The results of qRT-PCR analyses showed that *MT1JP* is expressed at higher levels in non-cancerous than in cancerous cell lines, consistent with the results from the tissue samples. Given that *MT1JP* had highest expression in the L02 cell line, we conducted the subsequent knock-down experiments in L02 cells, while over-expression experiments were carried out in the HepG2 and SMMC-7721 cell lines, which only express low level of endogenous *MT1JP*. All three cell lines are derived from liver tissues.

The lncRNA *MT1JP* resides on chromosome 16 in a cluster consisting of several homologous protein-coding genes of the metallothionein family. The *MT1JP* locus contains three regions that are highly conserved across a few of vertebrates, and is also annotated with several SNPs, CpG islands and clusters of DNase hypersensitive sites (Supplementary Figure S1C). Using 5′ and 3′ rapid amplification of cDNA ends (RACE) on RNA extracted from the L02 cell line, we showed that the mature *MT1JP* transcripts comes in two isoforms of 880 nt or 927 nt, both composed of two exons with a poly(A) tail attached (Figure 1D). The length of MT1JP we verified was nearly identical to one of the annotations in UCSC database (Figure 1D). In addition, northern blot analysis also identified *MT1JP* in the L02 cells, confirming the RACE results (Figure 1E). Since these two isoforms were almost identical, we did not distinguish each other when performing the subsequent experiments.

We then examined the coding potential of *MT1JP* using both *in silico* prediction and *in vivo* experiment. The *in silico* results obtained with four powerful computational tools for distinguishing coding and noncoding transcripts consistently showed that *MT1JP* has no ability to code a protein (Supplementary Table S1) [28–31] We nonetheless fused the only open reading frame (ORF) in the *MT1JP* transcript with *EGFP* (Figure 1F and Supplementary Figure S1D). By overexpressing the constructs in L02 cells, we found that while fused *MT1JP* ORF and *EGFP* could be expressed as a transcript, no fused protein was produced (Figure 1F). These results indicated that *MT1JP* is a long noncoding RNA which is down-regulated in cancer compared to adjacent normal tissues.

### *MT1JP* modulates the cell cycle

**Figure 2:**
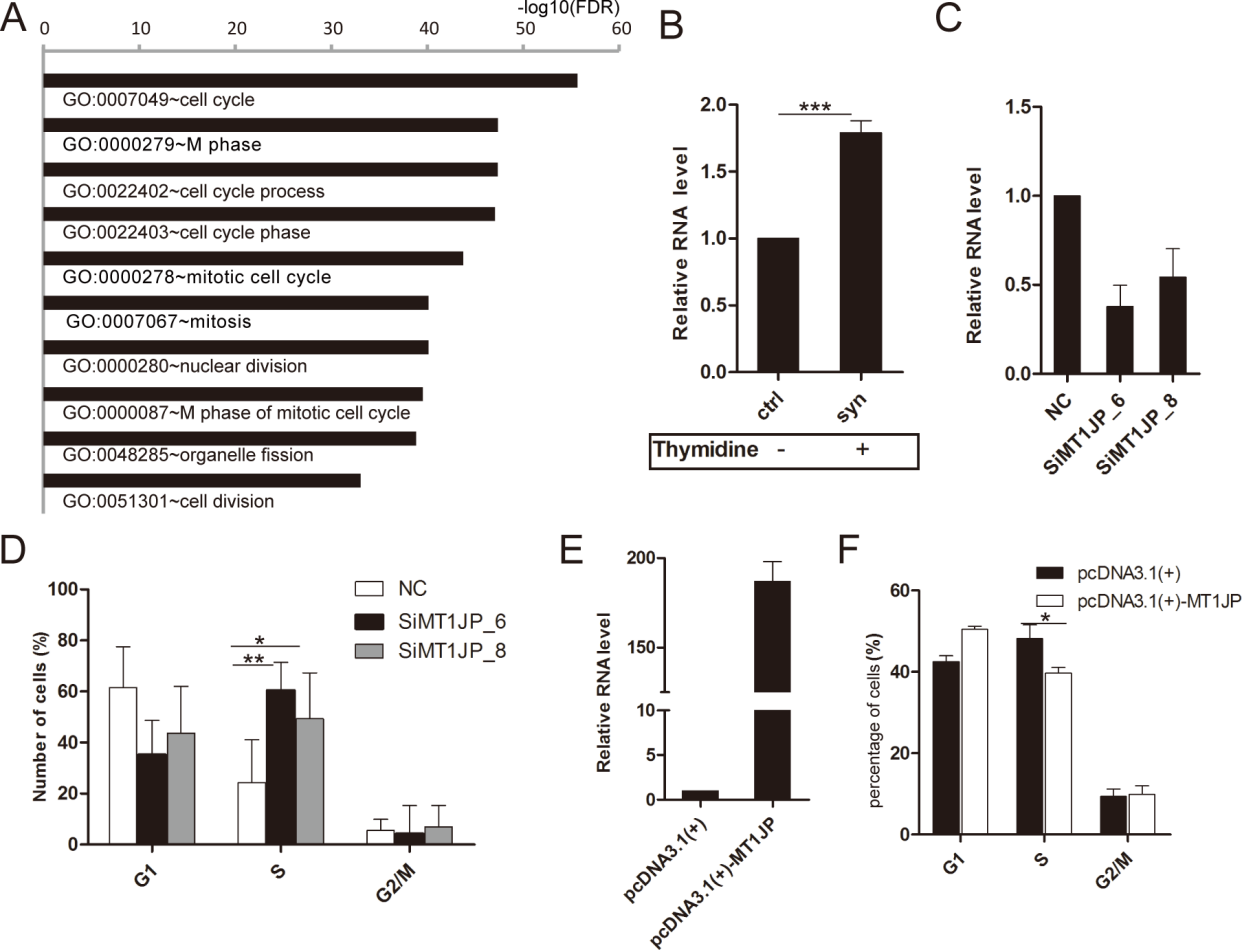
*MT1JP* acts as a cell cycle regulator (**A**) Significantly enriched gene ontology (GO) terms of genes whose expression are correlated with *MT1JP* in tumor samples. (**B**) Relative abundance of *MT1JP* was measured by qRT-PCR before and after treating cell with thymidine. (**C**) Targeting *MT1JP* in L02 cells by two independent siRNAs led to depletion of the transcript as measured by qRT-PCR. (**D**) Effect of *MT1JP* knock-down on the fraction of L02 cells in G1, S and G2/M cell cycle phases. (**E**) After transfection of cells with negative control or *MT1JP*-overexpressed vectors, *MT1JP* levels were measured as in (B). (**F**) Relative number of L02 cells in each phase of the cell cycle after treating cell as in (E). Values are the mean and error bars represent standard deviation (± SD) in triplicate experiments. *p < 0.05, **p < 0.01, ***p < 0.001.

To investigate which pathway *MT1JP* might be involved in, we conducted a Gene Ontology (GO) analysis of genes whose expression was strongly correlated (*r*^2^ > 0.25) to that of *MT1JP* in the normal and matched cancer tissues. The GO analysis identified significant enrichment for annotations related to the cell cycle (Figure 2A). Therefore, in order to explore the relationship between the cell cycle and the *MT1JP* expression level, we used the double thymidine method to synchronize the human hepatic cell line L02, and observed a significant increase in *MT1JP* expression after L02 cells were treated with double thymidine (Figure 2B). Furthermore, RNAi-mediated knockdown of *MT1JP* in L02 cells were performed with two different siRNA duplexes (Figure 2C). The down-regulation of *MT1JP* led to an increase in the proportion of cells in S phase and a decrease in G1 phase (Figure 2D). Inversely, overexpression of *MT1JP* in the liver hepatocellular carcinoma cell line HepG2 by transient transfection (Figure 2E) produced a higher percentage of cells in G1 phase but a lower percentage in S phase, when compared to control cells (Figure 2F), that is, the opposite phenotype of the knockdown experiment. Together, these results indicate that *MT1JP* has a significant effect on the G1/S check point, and that its ectopic expression can induce cell cycle arrest.

### *MT1JP* is a tumor suppressor candidate

Given the significantly differential expression of *MT1JP* between cancerous and normal tissue samples (Figure 1A), we speculated that this lncRNA might not only regulate the cell cycle but also play important roles in other pathways involving in cell transformation. Toward this end, we performed Gene Set Enrichment Analysis (GSEA) on the above mentioned microarray data in Figure 1A. The GSEA indicated that *MT1JP*-related genes were enriched for several signatures involved in various kinds of cancers (Supplementary Figure S2).

**Figure 3:**
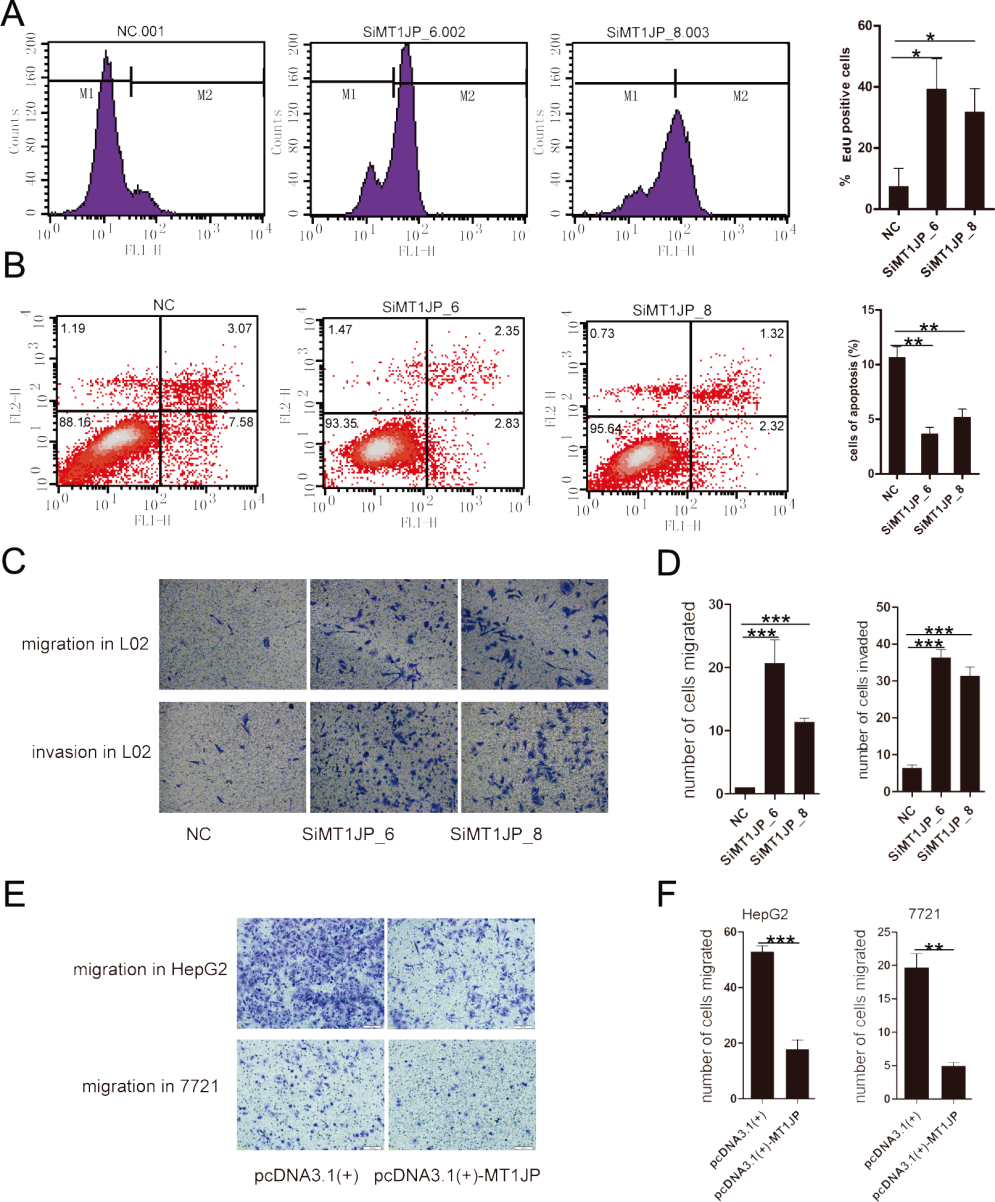
*MT1JP* is a potential tumor suppressor (**A**) BrdU labeling assay indicates that knockdown of *MT1JP* with siRNAs promotes cell proliferation. (**B**) Knockdown of *MT1JP* decreases the percentage of apoptotic cells. The percentage of cells in each quadrant is indicated. (**C** and **D**) Transwell assays indicate that *MT1JP* knockdown increases L02 cell migration and invasion. (**E** and **F**) Transwell assays as in (C and D) suggest that overexpression of *MT1JP* decreases cell migration. All values are means, and error bars represent standard deviation (± SD) of triplicate experiments. **p < 0.01, ***p < 0.001.

To address whether *MT1JP* exert its effects as a tumor suppressor, we first investigated in more detail the role of *MT1JP* in tumor suppression. The EdU (5-ethynyl-2′-deoxyuridine) incorporation assay was performed to investigate the effects of *MT1JP* on cell proliferation, and results showed that cells treated with *MT1JP*-targeting siRNA displayed stronger proliferation ability than cells treated with non-targeting siRNAs and ectopic expression of *MT1JP* decreased the EdU incorporation (Figure 3A and Supplementary Figure S3A). Next, we examined the impact of *MT1JP* knockdown on apoptosis in L02 and HepG2 cells by flow cytometry analysis and detecting cleaved-caspase3 which is a key representation of apoptotic cells. In line with our expectation, RNAi of *MT1JP* decreased the percentage of cells undergoing apoptosis while overexpression of *MT1JP* increased this percentage (Figure 3B and Supplementary Figure S3B). Collectively, the lncRNA *MT1JP* plays a potential role in promoting apoptosis to restrain the growth of aberrant cells. Additionally, we applied the transwell assay to examine alterations in L02 cell migration and invasion after knocking down *MT1JP*, and found that treatment of cells with *MT1JP* specific siRNAs increased the migration and invasive capacity of L02 cells when compared to ones with wild-type cells (Figure 3C and 3D). These results suggested that *MT1JP* has an inhibitory effect on proliferation, migration and invasion of L02 cells.

To further investigate the effects of *MT1JP* on tumor inhibition, we constructed a plasmid for overexpression of *MT1JP* in HepG2 and SMMC-7721 cells, which normally express MT1JP at lower levels than L02 cells. HepG2 overexpressing *MT1JP* cells were subjected to the transwell assay and their migration ability and invasive capacity were determined. It turned out that ectopic expression of *MT1JP* in HepG2 and SMMC-7721 cells reduced cellular migration capacitiy (Figure 3E and 3F). These gain-of-function results derived from the low expression cells complemented to the ones from knockdown experiments done in *MT1JP* high expressed cells, and indicated that *MT1JP* plays an important role in tumor migration etc. Altogether, these results provide evidence that *MT1JP* is a modulator of cell cycle, apoptosis, and proliferation, and may play a role as a tumor suppressor.

### *MT1JP* regulates the P53 signaling pathway

**Figure 4:**
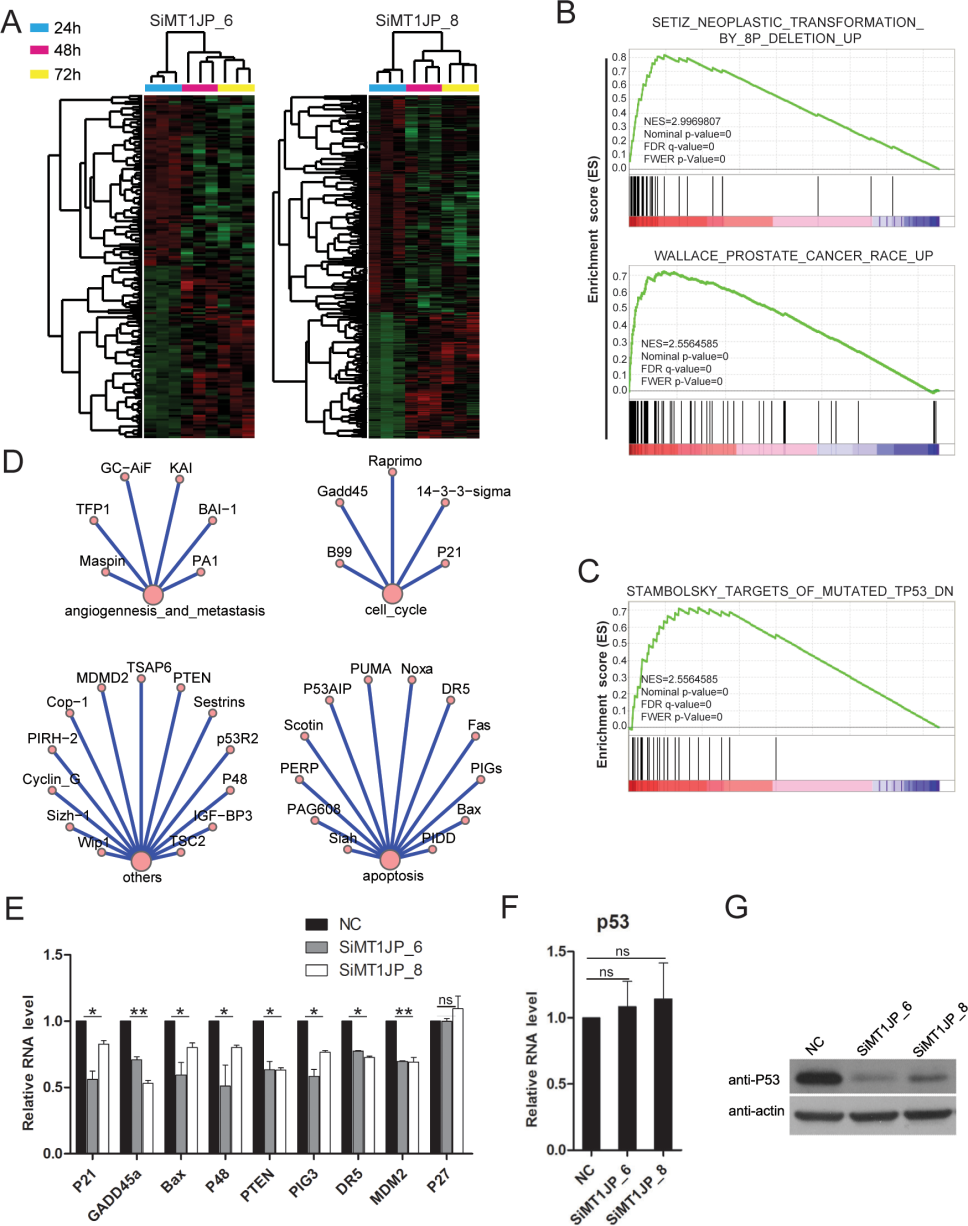
*MT1JP* regulates the p53 pathway (**A**) Heatmap illustration of *MT1JP* knockdown with two independent siRNAs after 24 h, 48 h and 72 h in L02 cells. Each knockdown has three replicates. (**B**) GSEA results show that the genes that differentially expressed (1.5-fold) after *MT1JP* knockdown in L02 cells are enriched for functional annotations related to cancer. (**C**) GSEA results showing significant enrichment of p53 targets signature for differentially expressed-genes in *MT1JP*-downregualted L02 cells. (**D**) TP53-associated pathways annotated in KEGG. (**E**) Effect of *MT1JP* knockdown on the expression of p53 target genes (qRT-PCR). (**F**) Effect of *MT1JP* knockdown on the p53 mRNA level (qRT-PCR). (**G**) Western blot analysis of the p53 protein level after *MT1JP* knockdown. All values are means and error bars represent standard deviation (± SD) in triplicate experiments. ns means not significant.

To elucidate the underlying molecular mechanisms of *MT1JP* function in the regulation of cell pathways, microarray analyses were performed to profile the transcriptome of L02 cells upon *MT1JP* knockdown. Two *MT1JP*-targeting siRNAs and negative control siRNA were transfected into L02 cells, and the cells were harvested after 24 hour (h), 48 h and 72 h separately. Transcriptome analyses showed major changes in the RNA expression profiles between 24 h and 48 h after transfection, but little changes beyond 48 h. In addition, we also identified 1650 genes (364 up-regulated and 1286 down-regulated) with more than 1.5-fold expression changes in the triplicate siRNA experiments 48 h after knockdown (Figure 4A and Supplementary Table S3). GSEA of these differentially expressed genes indicated enrichment for annotations related to neoplastic transformation, cancer and other tumor associated annotations (Figure 4B), which is consistent with the results as shown in Figures 2 and 3. In addition, the GSEA also showed that *MT1JP* had an effect on targets of p53, which belongs to the core of cell signaling pathway (Figure 4C). In the KEGG (Kyoto Encyclopedia of Genes and Genomes), p53 is annotated to be involved in apoptosis, cell cycle, angiogenesis, metastasis and other pathways (Figure 4D). To validate the finding that *MT1JP* regulates targets of p53, we transfected L02 cells with siRNAs targeting *MT1JP*, and determined the expression levels of several p53 targets by qRT-PCR. The experimental validation is consistent with the results of the GSEA, indicating that MT1JP regulates p53 target genes, for example *GADD45a* and p21, but has no effect on p27 which is not the member of p53 pathway (Figure 4E).

Given that *MT1JP* may play a role in regulating the expression of p53 downstream targets, we examined the effect of *MT1JP* knockdown on p53 expression both at the mRNA and protein levels. Interestingly, *MT1JP* knockdown resulted in a decrease in the p53 protein level but had no significant effect on its mRNA level (Figure 4F).

All together, these results suggest that *MT1JP* may facilitate expression of tumor suppressor p53 at the post-transcriptional, but not at the transcriptional level, and in turn affect the downstream genes of p53.

### *MT1JP* is largely cytoplasmic and interacts with TIAR

**Figure 5:**
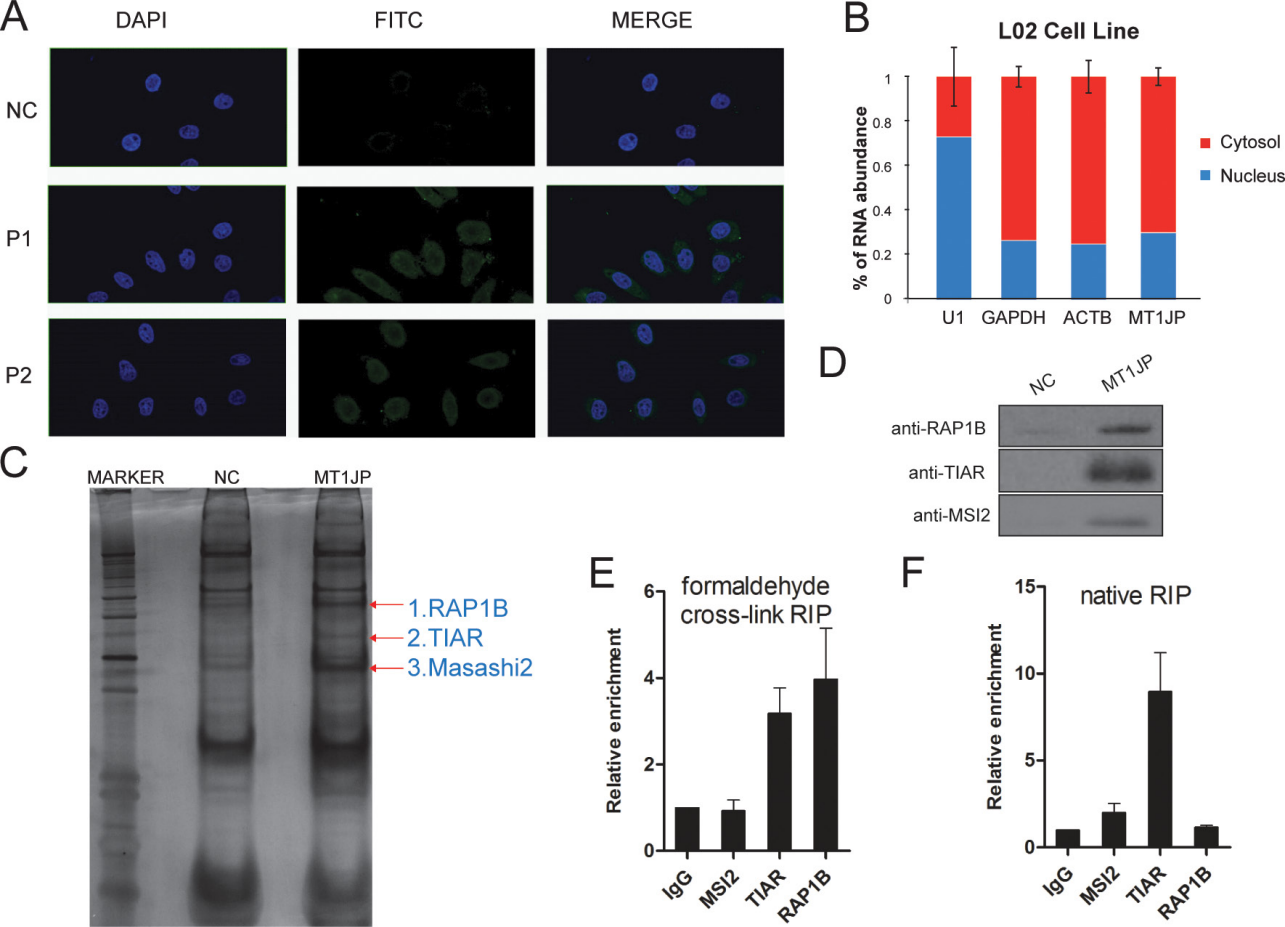
*MT1JP* is a cytoplasmic lncRNA which interacts with TIAR (**A**) RNA fluorescence *in situ* hybridization (FISH) of *MT1JP* in L02 cells with two independent LNA probes (P1 and P2) targeting *MT1JP*. (**B**) *MT1JP* subcellular localization is detected through cellular fractionation. The relative abundance in each component is determined by qRT-PCR. (**C**) RNA pull-down of *MT1JP*-associated proteins using biotinylated *MT1JP* or *lacZ* DNA probes. Isolated proteins were resolved by SDS-PAGE followed by silver staining. The highlighted bands were analyzed by mass spectrometry. (**D**) Western blot analysis of the specific interaction between candidate proteins and *MT1JP* with MSI2, TIAR and RAP1B antibody after RNA pull-down as in (B). (**E**) Association between *MT1JP* and candidate proteins in formaldehyde cross-link RIP by qRT-PCR. (**F**) Relative *MT1JP* enrichment in native RIP (qRT-PCR). The enrichment of *MT1JP* in F and G is normalized to *GAPDH* (versus IgG). All values are means and error bars represent standard deviation (± SD) in triplicate experiments.

We next tried to investigate the mechanism by which *MT1JP* regulates gene expression during cell transformation. We first determined the subcellular distribution of *MT1JP* by RNA fluorescence *in situ* hybridization (RNA-FISH), and found that most of the *MT1JP* transcripts were located in the cytoplasm (Figure 5A). Moreover, we confirmed this observation by quantifying the expression ratio of *MT1JP* in cytoplasmic and nuclear fractions with qRT-PCR. The results showed that approximately 70% of *MT1JP* transcripts were found in the cytoplasm (Figure 5B).

LncRNAs frequently exert their function through interactions and play roles through their association with proteins [22, 32, 33]. To test whether this might also be the case for *MT1JP*, we first sought to identify interacting proteins by RNA pull-down assay. Specifically, synthetic biotinylated *MT1JP* or LacZ antisense probes were incubated with L02 cell lysate, and co-precipitated proteins were then purified with streptavidin magnetic beads, followed by polyacrylamide gel electrophoresis and specifically enriched bands by *MT1JP* biotinylated probes were further analyzed by mass spectrometry. Three candidates were identified and two of them are Masashi 2 (MSI2) and TIA1-related protein (TIAR), both known to be RNA-binding proteins (Figure 5C), and the third one is Ras-related protein Rap-1b (RAP1B), a protein involved in the Ras pathway [34]. Further analysis by Western blot showed that MSI2, TIAR and RAP1B were pulled down with the *MT1JP* specific probe, but not with the control lacZ probe (Figure 5D). In addition, we found that the majority of TIAR were located in the cytoplasm by cell nucleocytoplasmic separation, which is similar with *MT1JP* (Supplementary Figure S4A).

To further validate the specificity of the association between *MT1JP* and the three identified proteins, we performed both native and formaldehyde cross-linked RNA immunoprecipitation (RIP) with antibodies against RAP1B, TIAR and MSI2. After antibodies had been incubated with the L02 cell lysate, RNA-protein complexes were precipitated with protein A/G beads, and associated RNAs were extracted. We verified the efficacy of the RIP by Western blot (Supplementary Figure S4B), and analyzed the enriched *MT1JP* content in each RIP sample by qRT-PCR. We observed that when using the formaldehyde cross-linking RIP approach, the enrichment of *MT1JP* in complexes precipitated with antibodies against TIAR and RAP1B was 3 and 5-fold more, respectively when compared with precipitates using control IgG (Figure 5E). In contrast, when using native RIP approach, in complexes precipitated with antibodies against TIAR and MSI2 the enrichment of *MT1JP* was 10 and 2-fold more, respectively, when compared with control IgG (Figure 5F). These results implied that TIAR interacts strongly with *MT1JP*, whereas RAP1B and MSI2 associate more weakly with *MT1JP*. Collectively, *MT1JP* interacts specifically with RNA-binding protein TIAR.

### *MT1JP* and TIAR regulates p53 at the post-transcriptional level

**Figure 6:**
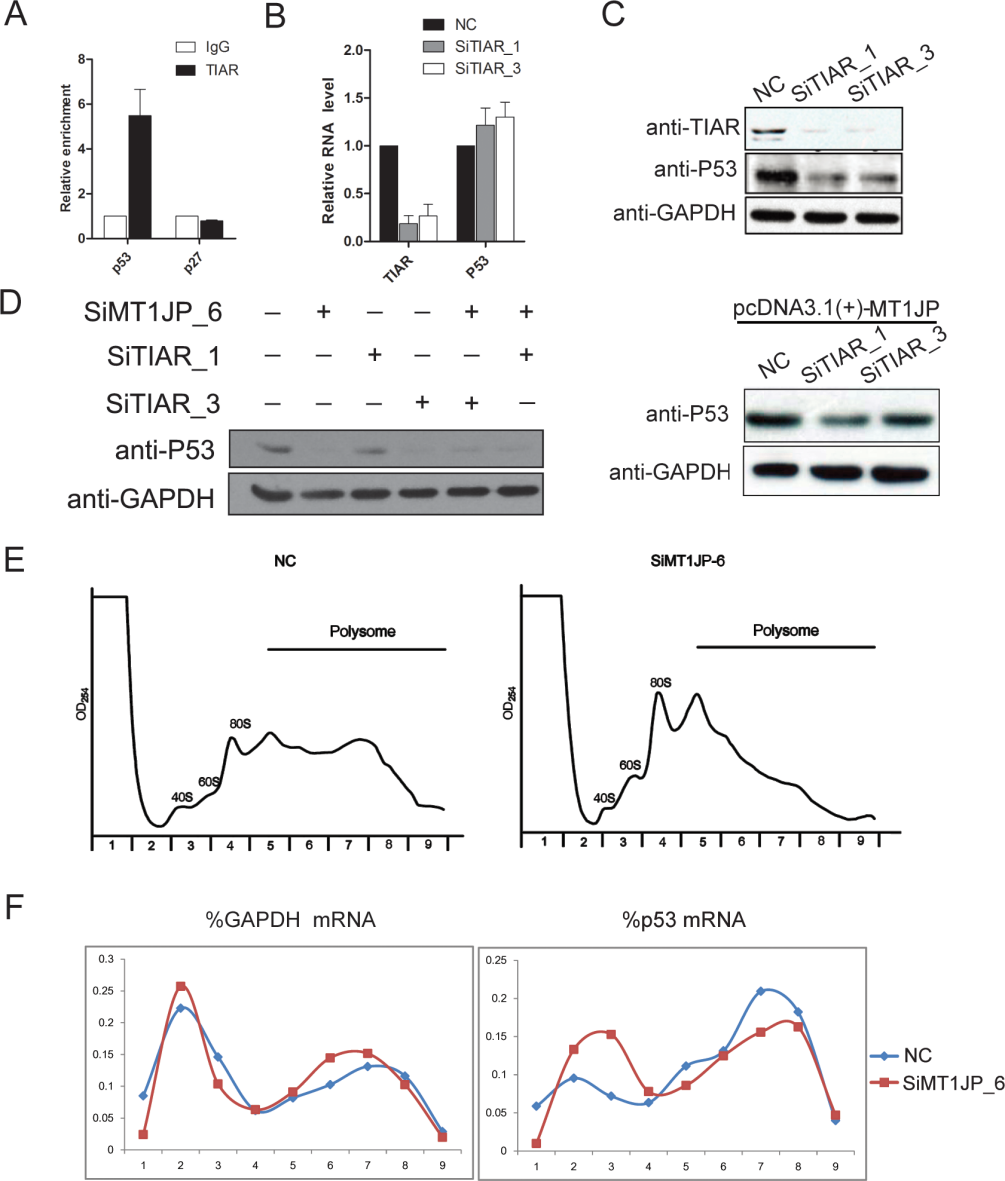
*MT1JP* and TIAR regulate p53 expression at the post-transcriptional level (**A**) Binding of p53 mRNA to TIAR in L02 cells, shown by RNA immunoprecipitation and qRT-PCR normalized with *GAPDH* (mean ± SD, *n* = 3, versus IgG). (**B**) Relative abundance of TIAR and p53 mRNAs was measured by qRT-PCR after TIAR knockdown (mean ± SD, *n* = 3). (**C**) Western blot analysis of the p53 protein level after TIAR knockdown. (**D**) Western blot analysis of the p53 protein level after *MT1JP* or/and TIAR knockdown. (**E**) Western blot analysis of the p53 protein level after MT1JP overexpression and TIAR knockdown. (**F**) Sucrose gradient fractionation of polysomes from L02 cells after *MT1JP* knockdown. (**G**) The polysomal distribution of the *p53* and *GAPDH* mRNAs was determined by qRT-PCR analysis of RNA in gradient fractions, and represented as percentage of total RNA in the fractions. Values represent mean of three independent experiments.

Several studies have suggested that a number of RNA-binding proteins are key factors in mRNA translational regulation [35, 36]. The data presented above further suggest that *MT1JP* interacts directly with TIAR and that *MT1JP* affects p53 expression, but not at the transcriptional level. Based on these indications, we speculated that *MT1JP* may form a complex with TIAR which affects the expression of p53 at the translational level. One possibility in such a model is that TIAR also interacts with the p53 mRNA. To test this speculation, we carried out TIAR RIP experiment and detected that the p53 mRNA was precipitated by an antibody against TIAR, whereas the negative control p27 mRNA was not (Figure 6A). To further validate our hypothesis, we treated L02 cell with two specific siRNAs to knockdown TIAR, and observed that down-regulation of TIAR had no effect on the p53 mRNA level (Figure 6B). However, knockdown of TIAR decreased the expression of the p53 protein (Figure 6C). Moreover, p53 protein was reduced when we knocked down *MT1JP* and TIAR simultaneously, which is consistent with knockdown of *MT1JP* and TIAR separately (Figure 6D). Nevertheless, the p53 protein could hardly be rescued by overexpression of MT1JP with TIAR repressed (Figure 6E). This result demonstrates that *MT1JP* regulates p53 protein by associating with TIAR.

To directly test the possibility that *MT1JP* promotes the translation of p53 mRNA, we utilized polysome fractionation to evaluate the translation status of p53 mRNA in L02 cells. Centrifuged through a 10%–50% sucrose gradient, the lightest components sedimented at the top (Fractions 1 and 2), ribosomal subunit (40S and 60S) and monosomes (80S) in fractions 3 and 4, smaller polysomes in fractions 5–6, and larger polysomes in fractions 7 and 9 (Figure 6E). The translational efficiency of an mRNA is reflected by its distribution within the sucrose gradient. Efficiently translated RNAs are associated with larger polysomes, and inefficiently translated RNAs with smaller polysomes or monosomes. After knockdown of *MT1JP*, the content of p53 mRNA in larger polysomes was reduced while it increased in smaller polysomes and non-polysomes, whereas the content of *GAPDH* mRNA in larger polysomes was increased (Figure 6F). Together, these results suggested that altering the *MT1JP* level affects the polysome distribution of p53 mRNA, thereby influencing p53 translational efficiency.

In addition, we also investigated the relationship between *MT1JP* and TIAR. Neither the TIAR mRNA nor the protein level was influenced by the knockdown of *MT1JP*. However, the expression of *MT1JP* was increased after knockdown of TIAR (Supplementary Figure S4A). TIAR has been reported as a factor controlling RNA turn-over [37], and we therefore treated L02 cells with the RNA polymerase II inhibitor α-amanitin. The *de novo* synthesis of *MT1JP* was blocked in L02 cells treated with α-amanitin. The knockdown of TIAR increased the stability of *MT1JP* when compared to control cells (Supplementary Figure S4B). All these data indicated that TIAR promotes *MT1JP* decay, thereby affecting the expression level of *MT1JP*.

## DISCUSSION

An increasing number of long noncoding RNAs has been discovered along with the advances in next-generation sequencing technology, however, our knowledge of their biological roles are limited. In this study, we identified a lncRNA *MT1JP* which appears to play a significant role in the inhibition of tumors. Our data show that *MT1JP* has considerably lower expression in tumor tissue samples than in matched normal tissue ones, which was observed among samples from gaster, colon, liver and lung. This consistent expression profile of lncRNA among different tissues has only been reported in a few cases. In cell lines from the same organs, the expression profiles of *MT1JP* were consistent with those in the tissues. Furthermore, knockdown of *MT1JP* resulted in a range of changes which usually arise during tumor formation, including cell cycle arrest, apoptosis inhibition, proliferation acceleration, and increased invasion and migration, as opposed to the effects of *MT1JP* overexpression. Down- or up-regulation of *MT1JP* led to variation in the expression of numerous cancer-associated mRNA molecules. These results suggest that *MT1JP* is necessary for maintaining the normal life activities of cells, and that it may have a critical function as a tumor suppressor.

Transcription factor p53 is a prominent tumor suppressor, and lncRNAs also have been reported to be involved in the p53 response as master regulators. *LincRNA-p21* is transcriptional target of p53, and acts as a repressor in the p53 regulation pathway to induce apoptosis [3], and the lncRNA *PANDA* is induced by p53 and plays a role in the cell cycle and in the apoptosis pathway [38]. In addition, lncRNA Pint is a bona fide p53 transcriptional effector and serves as a negative modulator of the p53 pathway for proliferation and apoptosis [6]. In contrast to these lncRNAs, *MT1JP* is not a transcriptional target of p53 (data not shown), but regulates the protein level of p53 through promoting the translation of its mRNA, thereby participating in all p53 pathways. The data presented here thus show that *MT1JP* acts as an upstream regulator of p53, and add to our understanding of lncRNA-associated gene modulation in the p53 pathway.

Previous studies have shown that a number of RNA-binding proteins can not only enhance but also suppress mRNA translation [39–42]. One such protein is HuR. The HuR protein was found to interact with 3′-UTR of the *Fas* mRNA to block its translation in liver cancer [43] while upregulation of HuR in human RKO colorectal carcinoma cells led to elevated p53 protein levels in response to ultraviolet light irradiation [44]. The RNA-binding protein TIAR is also a good example. TIAR is reported to have partially repressed global translation in tetracycline-inducible system, whereas a genome-wide profiling showed that TIAR upregulated p53 signaling pathway-related genes selectively [45]. In this study, we have shown that not only the RNA-binding protein TIAR but also the lncRNA *MT1JP* can upregulate p53 mRNA translation. TIAR interacts strongly with p53 mRNA and *MT1JP*, however, no direct interaction between *MT1JP* and p53 mRNA was detected (data not shown), which is explained by the lack of sequence complementarity between these two RNAs. Therefore, the modulation of p53 by *MT1JP* is TIAR-dependent, this is to say that the translational regulation of p53 occur by a complex of *MT1JP* and TIAR, in which TIAR interacts with the p53, but where the regulatory effect of *MT1JP* on p53 translation depends on the presence of TIAR in a fashion yet to be elucidated. Moreover, this is the first report showing TIAR can strongly bind to a lncRNA, and *MT1JP* is thus the first lncRNA found to act in concert with TIAR. Further experiments are required to confirm the detailed mechanism by which the *MT1JP*-TIAR complex acts on the p53 mRNA translation. Since *MT1JP* may have a role as a tumor suppressor in several kinds of cancers, it is likely that the translational regulation by the *MT1JP* protein complex demonstrated in this study is a widely occurring tumor suppression phenomenon. Even more importantly, the association between *MT1JP* and RNA-binding protein provides a novel node in the complicated cancer regulatory network.

We observed that in addition to TIAR, *MT1JP* can also bind to the RAP1B and MSI2 proteins. Given the known functional difference between these three proteins it seems reasonable to assume that *MT1JP* may play different role in the regulation of other genes or other cellular processes. It is reported that MSI2 can take part in post-transcriptional regulation through associating with specific mRNAs and that it may participate in the modulation of proliferation and maintenance of stem cells in the central nervous system [46, 47], and it has recently demonstrated a potential as a predictive biomarker of HCC prognosis [48]. RAP1B is a member of the RAS-related protein family, and is involved in a number of cellular processes including cell growth, differentiation, migration and invasion [49–51]. Although RAP1B has not been reported as a RNA-binding protein, our data suggest that it may add this feature to its functional repertoire.

**Figure 7:**
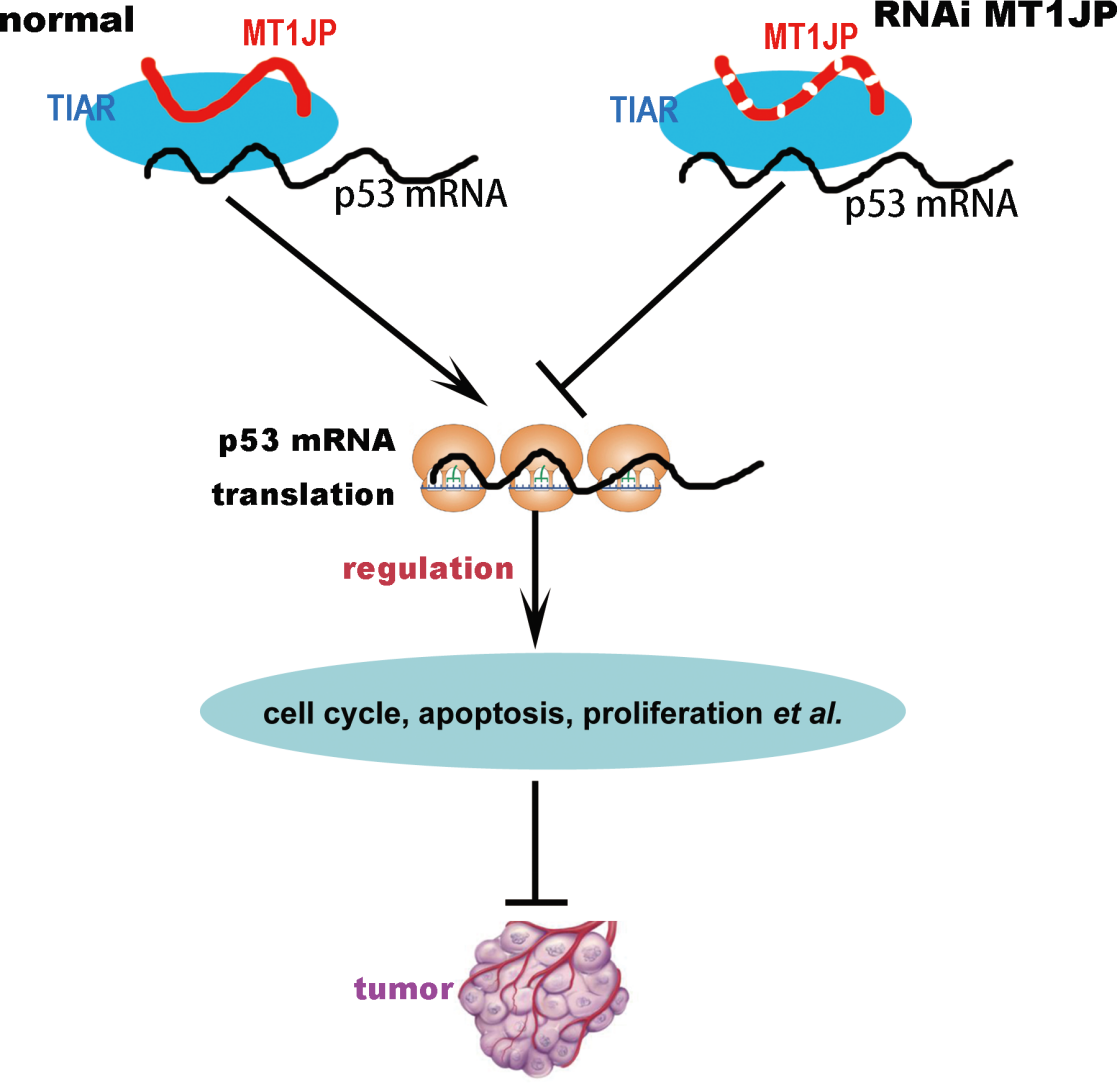
The model of lncRNA *MT1JP* regulating cell transformation

In summary, our results suggest that the cytoplasmic lncRNA *MT1JP* can bind diverse proteins with a potential to affect a variety of cellular processes. In its interaction with the RNA-binding protein TIAR, *MJ1JP* may function as a tumor suppressor through modulation of the p53 protein expression level and thereby the p53-related pathways (Figure 7).

## MATERIALS AND METHODS

### Cell culture

The human cell line L02 were cultured in RPMI 1640 Medium (ATCC Modification) and both SMMC-7721 and HepG2 cells were cultured in DMEM. All media were supplemented with 10% (v/v) FBS.

### Northern blotting

Northern blotting of MT1JP was performed according to Xiao *et al.* [52].

### Cell migration and invasion

For knockdown and overexpression of *MT1JP*, L02, SMMC-7721 or HepG2 cells were transfected with siRNAs or plasmids, trypsinized and counted after 24 h. 50 000 cells were seeded in the upper chamber of Transwell (Corning, #3422) with (for invasion) or without (for migration) BD Matrigel matrix (BD, #356230) coated. The medium in upper chamber has no fetal bovine serum (FBS) while the one in the lower chamber contains 20% FBS. After another 24 h culture at 37°C, 5% CO_2_, the non-migrated or non-invaded cells in upper chamber were gently removed, while the migrated or invaded cells were stained with 0.1% crystal violet for 20 min, washed with water, air dried and photographed using an Olympus microscope imaging systems. The migrated or invaded cells were counted for differential analysis.

### Subcellular fractionation

Cells were fractioned as NE-PER(R) Nuclear and Cytoplasmic Extraction's instruction (pierce, #78833). The total RNA isolated from each fraction was determined by qRT-PCR. *U1* serves as the nuclear control while *GAPDH* and *ACTB* as the cytosolic controls.

### RNA isolation and RT-qPCR

RNA was isolated with TRIzol Reagent (Invitrogen) according to manufacturer's instructions, and reverse transcribed using SuperScript^®^ III First-Strand Synthesis System (Invitrogen). QRT-PCR was performed with TransStart Top Green qPCR SuperMix (TransGen) according to the manufacturer's protocol. All the sequences of primers were in Supplementary Table S2.

### Western blotting

Western blotting was performed by standard protocols. Antibodies against the following protein were obtained as indicated: P53 (Senta Cruze), TIAR (Cell Signaling Technology), RAP1B (Proteintech), MSI2 (Abcam), GAPDH (Abnova).

### siRNAs, plasmids and cell transfection

siRNAs were designed by using webserver of Integrated DNA Technologies (http://www.idtdna.com). The full length *MT1JP* DNA sequence, potential ORF sequence and *EGFP* were cloned into pcDNA3.1(+) with double enzyme digestion reactions. For transfection of siRNAs or plasmids, L02, SMMC-7721 or HepG2 cells were cultured in 12-well or 10 cm plates overnight, and transfected with 20 uM siRNAs or plasmids using the Lipo2000 (Life Technology) kit according to the manufacturer's recommendations. All the sequences of primers, siRNAs and probes were in Supplementary Table S2.

### Silver stain

Silver staining was performed using the SilverQuest^™^ Silver Staining Kit Dynabeads Protein A (Invitrogen) according to the manufacturer's recommendations.

### RACE

5′ and 3′ RACE were performed using the FirstChoice RLM-RACE Kit (Life Technology) according to the manufacturer's recommendations. The primers are listed in Supplementary Table S2.

### Gene ontology (GO) analysis

The genes that have high correlation of expression with *MT1JP* in 76 pairs of tissue samples were analyzed to perform functional enrichment at webserver: http://david.abcc.ncifcrf.gov/tools.jsp.

### Bioinformatics analysis of microarray data

Feature Extraction v10.7.3.1 (Agilent Technologies, CA) software was used to extract all features of the data obtained from the scanned images. The signal ratio of Cy5 intensity and Cy3 intensity on the same array was subjected to quantile normalization followed by log2-scale transformation. Hierarchical clustering was performed using cluster 3.0 with complete linkage and centered Pearson correlation. The normalized and log2-scaled signal ratios were centered on the median before performing unsupervised hierarchical clustering.

### Statistical analysis

Experimental data are represented as the mean ± SD of more than three biologic replicates and Student's *t* tests were performed to determine the significance of differences. *P*-values are indicated as follows: *P < 0.05, **P < 0.01, and ***P < 0.001.

## SUPPLEMENTARY MATERIALS FIGURES AND TABLES




