# Supplementary material for: LncRNA MT1JP functions as a tumor suppressor by interacting with TIAR to modulate the p53 pathway
Source: Oncotarget. 2016 Feb 19;7(13):15787–800. doi: 10.18632/oncotarget.7487 (PMC4941277; doi:10.18632/oncotarget.7487)
Supplement: Supplementary file 1 [file oncotarget-07-15787-s001.pdf]

LncRNA MT1JP functions as a tumor suppressor by interacting with TIAR to modulate the p53 pathway

Supplementary Materials

Inventory

Supplementary Figure S1 (related to Figure 1: Identification of MT1JP as a lncRNA involved in tumorigenesis.
Supplementary Figure S2 (related to Figure 3: GSEA results of MT1JP-related genes in four kinds of tumor tissues showing significant enrichment of cancer-associated signatures.
Supplementary Figure S3 (related to Figure 3: MT1JP is a potential tumor suppressor.
Supplementary Figure S4 (related to Figure 5: The subcellular localization of TIAR and RNA immuno-precipitations (RIP) of three candidate proteins.
Supplementary Figure S5 (related to Figure 6: MT1JP is regulated by TIAR.

MATERIALS AND METHODS

RNA immunoprecipitation

Native RNA immunoprecipitation(RIP) was performed as follow: first, 5×10^6 cells were harvested for each reaction, trypsinated, washed with DEPC-PBS three times, resuspended in 1 ml IP lysis buffer (Pierce, #87788), and added 1% Proteinase inhibitor, 5‰ Rnasin(40U/ul), and 1‰ DTT(1M), and subsequently kept on ice for 30 min. The lysate was centrifuged at ~20000 g and 4°C for 20 minutes to pellet the cell debris, and the supernatant transferred to new tubes. Meanwhile, proteinA/G magnetic beads (Pirece, #88803) (100 ul for each reaction) were washed with lysis buffer three times. Then the lysate was precleared with half of the washed beads for 1 hour at 4°C, the beads discarded and the supernatant transferred to new tubes. After preclearing, antibody (1–10 ug for 1 reaction) was added to the precleared lysate and incubated overnight at 4°C. Followed that, the remaining beads were added to antibody-lysate mixture and incubated for 3 hours at 4°C. After incubation, the magnetic beads were washed with lysis buffer 3–5 times. Finally, add SDS protein loading buffer or TRIzol to the beads for SDS-PAGE or qRT-PCR.

Formaldehyde cross-link RIP was performed as reported by Zhang [1].

RNA-pull down

Briefly, 5×10^7 cells were harvested by trypsinization, washed with DEPC-PBS three times, resuspended in 5–10 ml IP lysis buffer (Pierce, #87788), added 1% Proteinase inhibitor, 5‰ RNasin and 1‰ DTT, and kept on ice for 30 min. The lysate was centrifuged at ~20000 g and 4°C for 20 minutes to pellet the cell debris, and the supernatant transferred to new tubes. Dynabeads™ T1 Streptaviden magnetic beads (Invitrogen, #65602)(60 ul for 1 ml lysate) were washed with Solution A (DEPC-treated 0.1 M NaOH and 0.05 M NaCl) for two times and Solution B (DEPC-treated 0.1 M NaCl) for one time, then resuspended in Solution B. Then the lysate was precleared with half of the washed beads for 1 hour at 4°C, the beads discarded and the supernatant transferred to new tubes. After preclearing, biotin labeled RNA or DNA probes were added to the precleared supernatant and incubated at 4°C for 3 hours with gentle agitation. After incubation, the remaining beads were added into the mixture in the last step, incubated at 4°C for another 3 hours. The resultant RNA-protein complexes were then eluted with magnetic bead binding, the solution could be discarded (or saved as input) and the beads (putatively bound with the RNA-protein complexes) washed 5 times with wash buffer (10 mM Tris pH 8.0, 1 mM EDTA, 0.5 M NaCl). Finally, add SDS protein loading buffer or TRIzol to the beads for SDS-PAGE or qRT-PCR.

Fluroscence *in situ* hybridization (FISH)

The cells of interest were plated on sterile glass coverslips the day before the experiment. The next day, the cells were washed with PBS and fixed for 10 min with 4% PFA at RT. Then the cells were incubated with 1 ml of cold permeabilization buffer for 5 min at 4°C. Before adding probes to the hybridization buffer and applying to the coverslips, the prehybridization was performed for 20 min at 22–25°C below the predicted Tm value of the LNA probes (Exiqon), followed by hybridization for 1 h at the same temperature. Then the cells were washed to remove redundant probes with washing buffer. After that, the cells were incubated with 3% H_2O_2, for 20 min at room time, and then washed with TN buffer. To reduce non-specific binding, the coverslips were incubated in TNB blocking buffer for 30 min at RT, then added anti-DIG antibody and Hoechst (dilute in TNB buffer) and incubated for 30 min at RT. At last, added TSA solution and incubated for 10 min at RT and washed three times with TNT buffer. Pictures were taken with OLYMPUS FV1000.

Cell proliferation, apoptosis and cell cycle analysis

Analysis of cell proliferation were carried out with the Click-iT® EdU Alexa Fluor® 488 Imaging Kit (Invitrogen), and cell apoptosis were detected using the FITC Annexin V/Dead Cell Apoptosis Kit with FITC annexin V and PI for Flow Cytometry (Invitrogen) according to the manufacturer›s recommendations. For cell cycle analysis, cells were fixed with 75% ethanol, then stained with propidium iodide (PI) for 30 minutes at 4°C, and quantified with a BD FACS Calibur flow cytometer (BD Biosciences).

Double thymidine block

Cell cycle synchronization was carried out with double thymidine as reported by Bostock *et al* [2].

Polysome analysis

Poysome fractionations were assayed according to the JoVE protocol [3]. After isolation, the RNA in each fraction was extracted and the relative abundance of p53 and GAPDH mRNAs were determined by qRT-PCR.

Bioinformatics analysis of microarray data

Feature Extraction v10.7.3.1 (Agilent Technologies, CA) software was used to extract all features of the data obtained from the scanned images. The signal ratio of Cy5 intensity and Cy3 intensity on the same array was subjected to quantile normalization followed by log2-scale transformation. Hierarchical clustering was performed using cluster 3.0 [4] with complete linkage and centered Pearson correlation. The normalized and log2-scaled signal ratios were centered on the median before performing unsupervised hierarchical clustering.

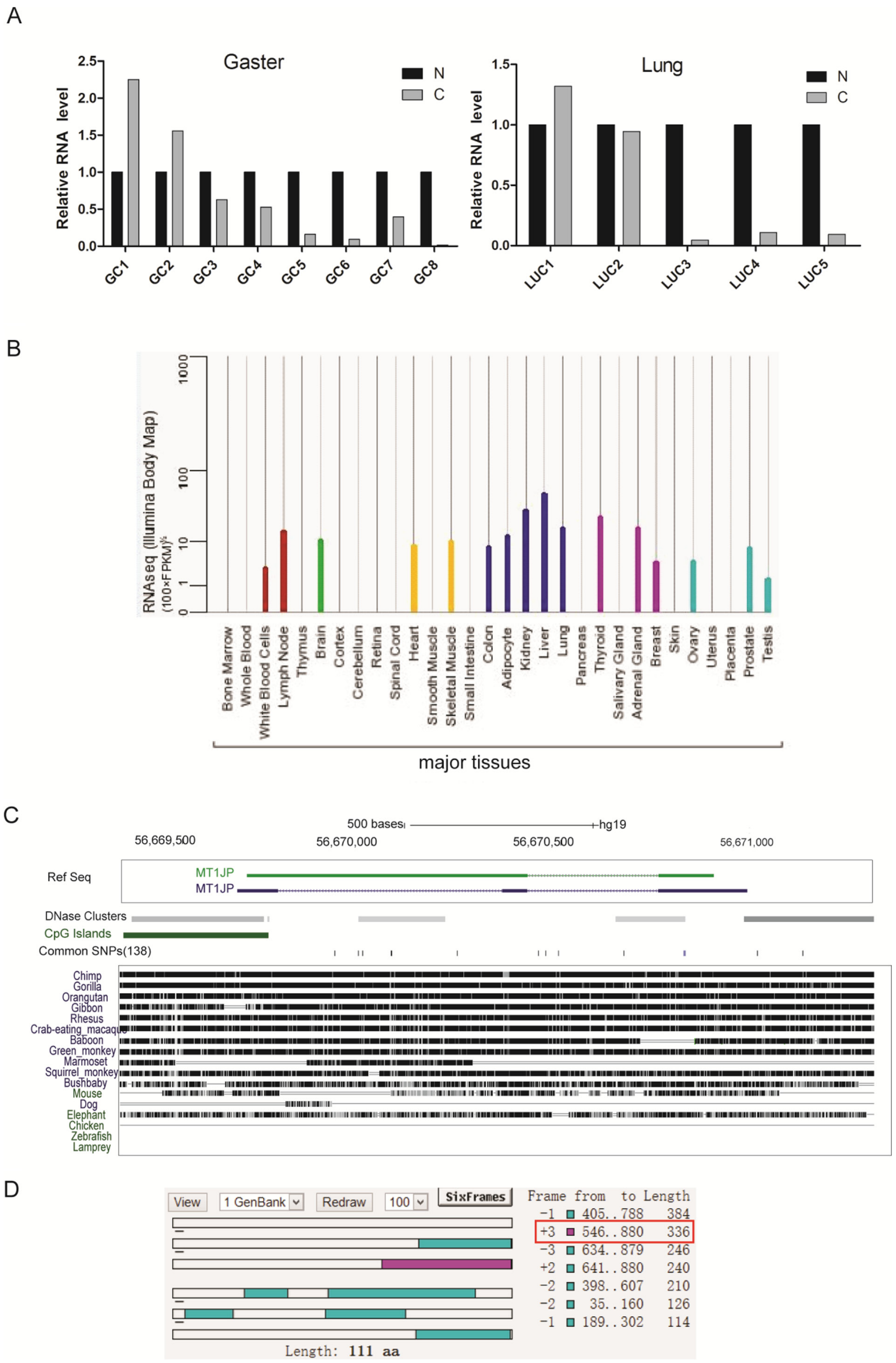

Supplementary Figure S1: Identification of MT1JP as a lncRNA involved in tumorigenesis. (**A**) Relative expression of *MT1JP* in a separate 16 pairs of normal (N) and cancer (C) tissue samples from gaster and lung were determined by qRT-PCR. (**B**) The data from human body map show that MT1JP expresses in different kinds of tissues. (**C**) Schematic representation of the MT1JP locus. (**D**) ORF finder shows that there is one open read frame candidate (highlighted).

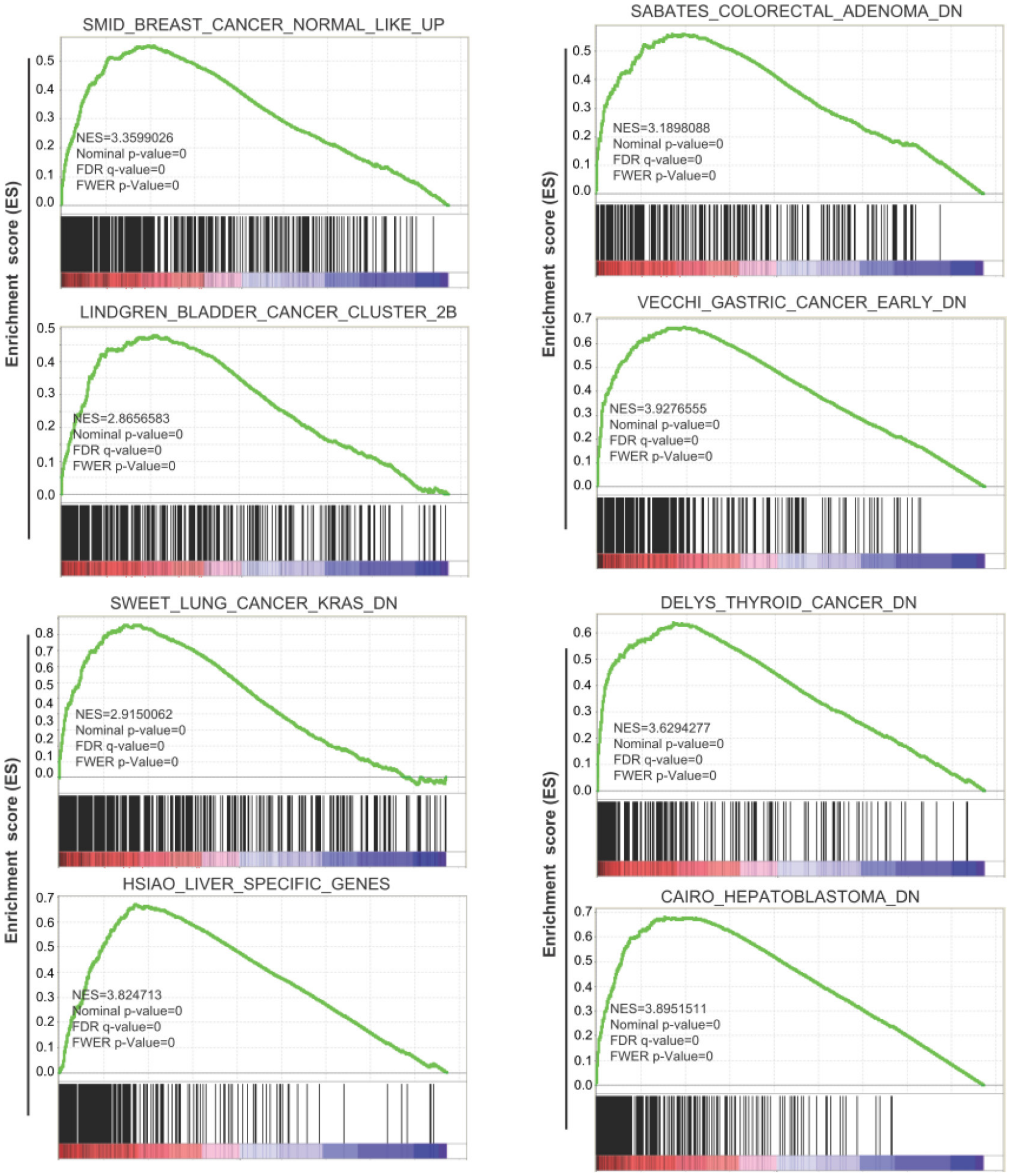

Supplementary Figure S2: GSEA results of MT1JP-related genes in four kinds of tumor tissues showing significant enrichment of cancer-associated signatures.

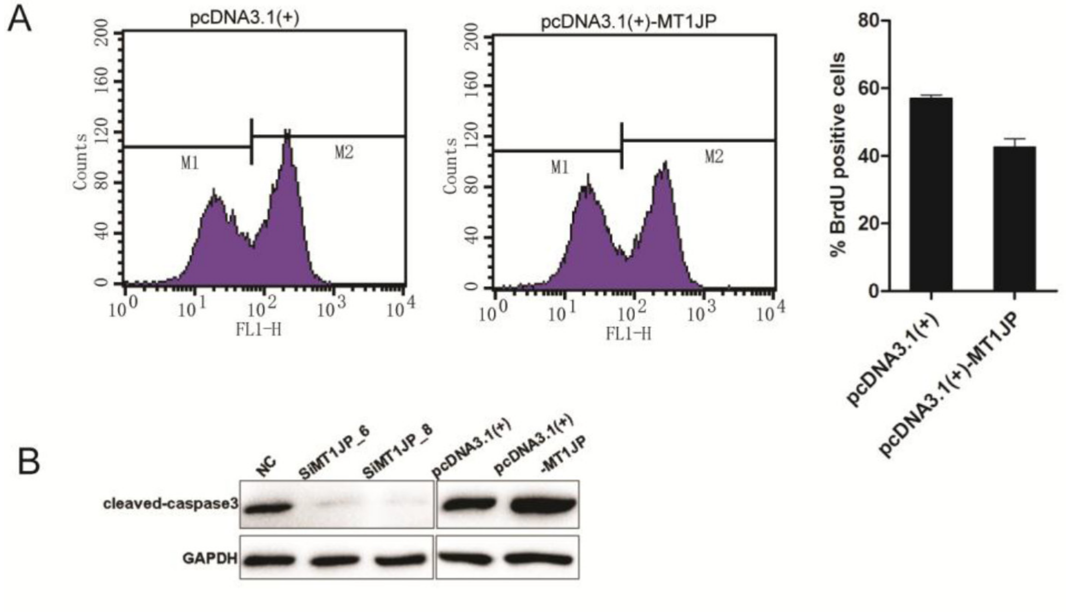

Supplementary Figure S3: *MT1JP* is a potential tumor suppressor. (**A**) BrdU labeling assay indicates that ectopic expression of *MT1JP* inhibits the cell proliferation. (**B**) Western blot analysis of the cleaved-caspase3 protein level after *MT1JP* knockdown or overexpression.

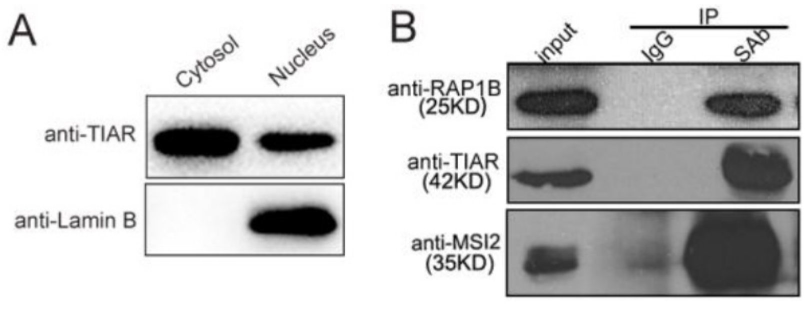

Supplementary Figure S4: The subcellular localization of TIAR and RNA immunoprecipitations (RIP) of three candidate proteins. (**A**) TIAR subcellular localization is detected after cellular fractionation. As a control, Lamin B is located in nucleus. (**B**) RNA immunoprecipitations (RIP) of three candidate proteins were analyzed by western blot with each antibody (The antibodies used from top to down are RAP1B, TIAR and MSI2 respectively). Sab is the abbreviation of specific antibodies.

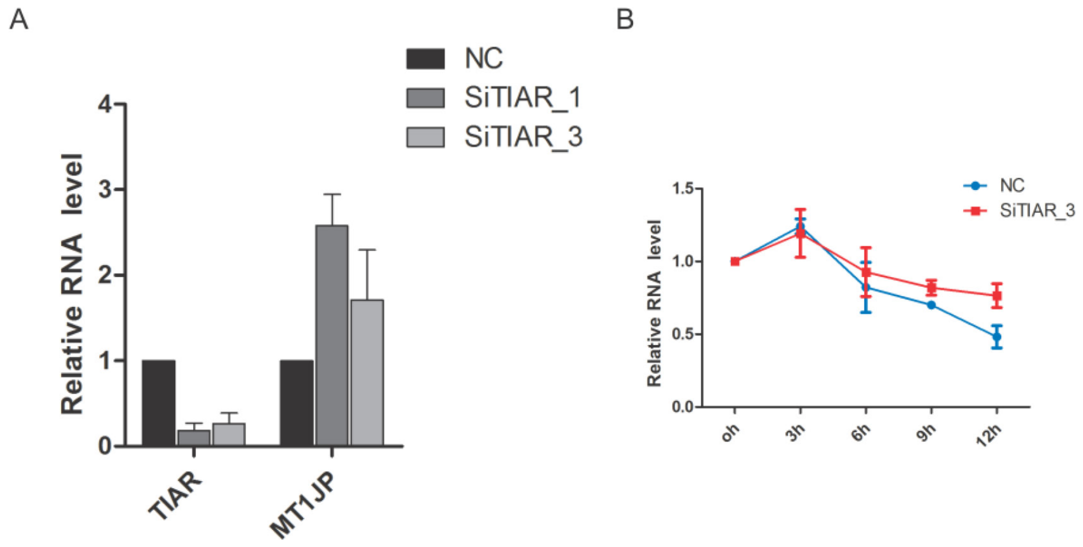

Supplementary Figure S5: MT1JP is regulated by TIAR. (**A**) The relative abundance of MT1JP was measured by RT-qPCR after knockdown of TIAR with two independent siRNAs. (**B**) The relative abundance of MT1JP was measured by RT-qPCR after α-amanitin treatment in control and MT1JP-downregulated cells.

Supplementary Table S1: The coding potential of MT1JP

Prediction tool	Coding potential	Score
CPC	Non-coding	–0.688839
CNCI	Non-coding	NA*
PhyloCSF	Non-coding	–7.9498
CPAT	Non-coding	NA*

*NA means no score produced.

Supplementary Table S2: Primers, siRNAs and probes used in this work

Primer	Sequence	Application
MT1JP_q-F	GGGACTCCTTTACTTCCTTGG	qPCR
MT1JP_q-R	CCTTGAGCCTCAGTATCCTTAAC	qPCR
p53_q_F	GGAAATTTGCGTGTGGAGTATTT	qPCR
p53_q_R	GTTGTAGTGGATGGTGGTACAG	qPCR
GADD45-q-F	AGAAGACCGAAAGGATGGATAAG	qPCR
GADD45-q-R	AGGCACAACACCACGTTAT	qPCR
MDM2-q-F1	AGGCTGATCTTGAACTCCTAAAC	qPCR
MDM2-q-R1	CAGGTGCCTCACATCTGTAATC	qPCR
DR5-q-F	AGTGTGTCAGTGCGAAGAAG	qPCR
DR5-q-R	CAATCACCGACCTTGACCAT	qPCR
Bax-q-F	TTCTGACGGCAACTTCAACT	qPCR
Bax-q-R	CAGCCCATGATGGTTCTGAT	qPCR
PTEN-q-F	ATGTGGCGGGACTCTTTATG	qPCR
PTEN-q-R	GCGGCTCAACTCTCAAACT	qPCR
P48-q-F	CTCCAGAGCTCCGACTTATTTC	qPCR
P48-q-R	GCACACACTCCTATGCTATCTC	qPCR
PIG3-q-F	GGCTGGAGACTATGTGCTAAT	qPCR
PIG3-q-R	CAGCTGTGACCAGAGGAATAG	qPCR
P21-q-F	ATGAGTTGGGAGGAGGCA	qPCR
P21-q-R	CAAGGGTACAAGACAGTGACAG	qPCR
P27-q-F	CTCGACTCAGACGTGGATAAAG	qPCR
P27-q-R	TCCGGACGAGGTACTGG	qPCR
ACTB_F	GGCCGAGGACTTTGATTGCACATT	qPCR
ACTB_R	AGGATGGCAAGGGACTTCCTGTAA	qPCR
hGAPDH_F1	TGCCATCAATGACCCCTTC	qPCR
hGAPDH_R1	CATCGCCCCACTTGATTTTG	qPCR
hU1_F	ATACTTACCTGGCAGGGGAG	qPCR
hU1_R	CAGGGGAAAGCGCGAACGCA	qPCR
TIAR-q-F1	CAGTCAGATTGGACCCTGTAAA	qPCR
TIAR-q-R1	AGCTGCATCTCTGTGTTCATAA	qPCR
RACE5_outer	CCTTGAGCCTCAGTATCCTTAAC	PCR
RACE5_inner	CCAAGGAAGTAAAGGAGTCCC	PCR
RACE3_outer	GGGACTCCTTTACTTCCTTGG	PCR
RACE3_inner	GTTAAGGATACTGAGGCTCAAGG	PCR
MT1JP_F_BamH1	TAGGATCCCGGTCTCTCCATTTATCGCTTGAG	PCR
MT1JP_R_EcoR1	TA GAATTCAGGTTGTGCAGGTTGTTCTAT	PCR
EGFP-EcoR1-F	ATACCGGAATTCATGGTGAGCAAGGGCGAG	PCR

EGFP-Xho1-R	TATCCGCTCGAGTCACTTGTACAGCTCGTCCATG	PCR
ORF-F_BamH1	CGGGATCCCTGAGTGGGAAAGGAGCTCTGAGGG	PCR
ORF-R_EcoR1	CGGAATTCTCCAGGTTGTGCAGGTTGTTCTATTAAC	PCR
SiMT1JP_6_sense	rGrGrCrArUrUrGrArGrUrCrUrUrCrArGrGrGrUrUrCrArGGA	SiRNA
SiMT1JP_6_antisense	rUrCrCrUrGrArArCrCrCrUrGrArArGrArCrUrCrArArUrGrCrCrUrC	SiRNA
SiMT1JP_8_sense	rCrArCrCrCrArGrUrUrGrGrUrCrArGrGrGrUrCrCrUrGrCTG	SiRNA
SiMT1JP_8_antisense	rCrArGrCrArGrGrArCrCrCrUrGrArCrCrArArCrUrGrGrGrUrGrArU	SiRNA
SiTIAR_1_sense	rGrGrArGrGrArArUrUrGrCrGrUrCrUrGrGrGrUrUrArArCAG	SiRNA
SiTIAR_1_antisense	rCrUrGrUrUrArArCrCrCrArGrArCrGrCrArArUrUrCrCrUrCrCrArC	SiRNA
SiTIAR_3_sense	rArCrArCrUrArArGrCrArGrUrUrGrArGrArUrUrUrGrArAGA	SiRNA
SiTIAR_3_antisense	rUrCrUrUrCrArArArUrCrUrCrArArCrUrGrCrUrUrArGrUrGrUrUrG	SiRNA
LacZ_PD1	GCCAGTGAATCCGTAATCAT	pull down
LacZ_PD2	AATGTGAGCGAGTAACAACC	pull down
LacZ_PD3	GATCTTCCAGATAACTGCCG	pull down
LacZ_PD4	GCTCATCGATAATTTCACCG	pull down
LacZ_PD5	TTAACGCCTCGAATCAGCAA	pull down
LacZ_PD6	ATTTGATCCAGCGATACAGC	pull down
LacZ_PD7	GGGTTGCCGTTTTCATCATA	pull down
LacZ_PD8	CACTTACGCCAATGTCGTTA	pull down
MT1JP_PD1	AGGAGCGCGATCAGGAAGAAAGCAC	pull down
MT1JP_PD2	TGCCTCTAGCTCAGCATCCACCCTG	pull down
MT1JP_PD3	AGCCTCAGTATCCTTAACTC	pull down
MT1JP_PD4	CAATGCCTCTAGCTCAGCAT	pull down
MT1JP_PD5	AAAACGCAGTTGGCAGTGAG	pull down
MT1JP_PD6	CTCCTGTTTCACCTTATCAG	pull down
MT1JP_LNA_1	AATGCCTCTAGCTCAGCATCCA	FISH and northern
MT1JP_LNA_2	TCACACTGGGAAGGGCAAGCA	FISH

Supplementary Table S3: Differential expression genes with more than 1.5 fold changes after knockdown MT1JP

REFERENCES

1. Zhang Y, Zhang XO, Chen T, Xiang JF, Yin QF, Xing YH, Zhu S, Yang L, Chen LL. Circular intronic long noncoding RNAs. Mol Cell. 2013; 51:792–806.
2. Bostock CJ, Prescott DM, Kirkpatrick JB. An evaluation of the double thymidine block for synchronizing mammalian cells at the G1-S border. Exp Cell Res. 1971; 68:163–168.
3. Gandin V, Sikstrom K, Alain T, Morita M, McLaughlan S, Larsson O, Topisirovic I. Polysome fractionation and analysis of mammalian translatomes on a genome-wide scale. J Vis Exp. 2014.
4. Eisen MB, Spellman PT, Brown PO, Botstein D. Cluster analysis and display of genome-wide expression patterns. Proc Natl Acad Sci U S A. 1998; 95:14863–14868.
